# Investigating Acoustic and Psycholinguistic Predictors of Cognitive Impairment in Older Adults: Modeling Study

**DOI:** 10.2196/54655

**Published:** 2024-09-16

**Authors:** Varsha D Badal, Jenna M Reinen, Elizabeth W Twamley, Ellen E Lee, Robert P Fellows, Erhan Bilal, Colin A Depp

**Affiliations:** 1 Department of Psychiatry University of California San Diego San Diego, CA United States; 2 Sam and Rose Stein Institute for Research on Aging University of California San Diego San Diego, CA United States; 3 IBM Research Yorktown Heights, NY United States; 4 VA San Diego Healthcare System San Diego, CA United States

**Keywords:** acoustic, psycholinguistic, speech, speech marker, speech markers, cognitive impairment, CI, mild cognitive impairment, MCI, cognitive disability, cognitive restriction, cognitive limitation, machine learning, ML, artificial intelligence, AI, algorithm, algorithms, predictive model, predictive models, predictive analytics, predictive system, practical model, practical models, early warning, early detection, NLP, natural language processing, Alzheimer, dementia, neurological decline, neurocognition, neurocognitive disorder

## Abstract

**Background:**

About one-third of older adults aged 65 years and older often have mild cognitive impairment or dementia. Acoustic and psycho-linguistic features derived from conversation may be of great diagnostic value because speech involves verbal memory and cognitive and neuromuscular processes. The relative decline in these processes, however, may not be linear and remains understudied.

**Objective:**

This study aims to establish associations between cognitive abilities and various attributes of speech and natural language production. To date, the majority of research has been cross-sectional, relying mostly on data from structured interactions and restricted to textual versus acoustic analyses.

**Methods:**

In a sample of 71 older (mean age 83.3, SD 7.0 years) community-dwelling adults who completed qualitative interviews and cognitive testing, we investigated the performance of both acoustic and psycholinguistic features associated with cognitive deficits contemporaneously and at a 1-2 years follow up (mean follow-up time 512.3, SD 84.5 days).

**Results:**

Combined acoustic and psycholinguistic features achieved high performance (*F*_1_-scores 0.73-0.86) and sensitivity (up to 0.90) in estimating cognitive deficits across multiple domains. Performance remained high when acoustic and psycholinguistic features were used to predict follow-up cognitive performance. The psycholinguistic features that were most successful at classifying high cognitive impairment reflected vocabulary richness, the quantity of speech produced, and the fragmentation of speech, whereas the analogous top-ranked acoustic features reflected breathing and nonverbal vocalizations such as giggles or laughter.

**Conclusions:**

These results suggest that both acoustic and psycholinguistic features extracted from qualitative interviews may be reliable markers of cognitive deficits in late life.

## Introduction

It is estimated that approximately one-third of adults aged 65 years and older in the United States have mild cognitive impairment (MCI) or dementia [1]. Given the high prevalence of MCI and dementia, better methods are needed for earlier identification. Biomarkers associated with future cognitive decline can be evident decades before the deficits are clinically detected [2]. By the time cognitive changes are evident to the patient or their families, functional difficulties may be more advanced, and potential interventions to reduce decline may be less effective.

Language abilities remain largely preserved in typical aging, despite gradual declines in other cognitive functions such as processing speed. The decline in language abilities may serve as a key indicator of atypical or pathological aging such as MCI and dementia [3-5]. Prior studies have reported speech and language declines across disease progression from early MCI to moderate-stage Alzheimer disease [6-8]. Mueller et al [8] found that subtle declines in speech fluency and semantic content are apparent even prior to the onset of clinically diagnosed MCI. Therefore, developing techniques to identify early changes in language functioning may enhance the detection of subtle cognitive decline associated with pathological cognitive aging.

Natural language processing (NLP) has emerged as a promising approach for identifying early signs of pathological cognitive decline [9]. NLP includes a variety of techniques to capture and quantify linguistic or semantic aspects of speech (eg, syntactic complexity, idea density, and semantic content) and is often combined with machine learning (ML) to automate classification based on latent patterns in data. Asgari et al [10] used linguistic features derived from audio recordings of unstructured conversations with 41 older adults and were able to differentiate cognitively intact individuals from participants with MCI with 84% classification accuracy. Studies using intermittent audio recordings of older adults without cognitive impairment in real-world settings over 4 days found that several linguistic features were associated with performance on standardized measures of working memory [11,12]. In a longitudinal analysis of data from the Framingham Heart Study, linguistic variables derived from written responses to a picture description task (the cookie theft task), improved the predictive accuracy of conversion from cognitively normal to Alzheimer disease over nonlinguistic markers [13]. Though transcripts have been the current standard of understanding language, audio features remain underresearched in psychiatry. Another possibility is that audio feature files are difficult to share due to privacy controls, and therefore, are more difficult to access. Acoustic features of speech (eg, volume) have been evaluated to a lesser extent than linguistic features [14-16]. To date, few studies evaluated the relative predictive performance of acoustic versus psycholinguistic-derived features, and none, to our knowledge, have evaluated performance in predicting future cognitive deficits.

This study aimed to explore and identify acoustic and psycholinguistic features associated with cognitive abilities on neuropsychological measures, both contemporaneously and at follow-up assessments, among older adults recruited from an independent living facility. Our work is unique from prior research in several ways. First, we use both acoustic and psycholinguistic features from speech derived from a semistructured interview rather than using standardized tasks. Second, all participants underwent a research-based neuropsychological assessment comprising multiple cognitive domains (eg, global cognitive ability as measured by a screening instrument, verbal memory, and phonemic and semantic fluency). Third, we collected follow-up neuropsychological data to evaluate the stability of prediction. Finally, we used ML techniques to develop predictive algorithms to identify acoustic and psycholinguistic features associated with cognitive impairment and change, using sociodemographic features alone as a comparison. We hypothesized that both acoustic and psycholinguistic features derived from the recordings of semistructured interviews would predict baseline and follow-up cognitive deficits, exceeding the predictive accuracy of sociodemographic characteristics (eg, age). We explored the contribution of individual features to these predictions and the relative performance of acoustic and psycholinguistic feature-based models across cognitive domains.

## Methods

### Participants and Procedures

This is a secondary analysis of interview transcripts from a sample that was previously described [17,18]. The original study goals were to examine predictors of cognitive and functional decline in a community-dwelling sample of older adults; thus, the lower age limit was set at 65 years in order to be representative of older adult populations that are at risk for cognitive decline. There was no upper age limit for inclusion in this study [17]. Participants were recruited from the independent living sector of a continuing care senior housing community in San Diego County. Enrollment criteria were (1) English-speaking individuals aged 65 years or older, (2) ability to complete study assessments and engage in a qualitative interview, and (3) no known diagnosis of dementia or any other disabling illness. For the study, we excluded individuals with medical or neurological conditions that would impede their ability to complete the assessments. However, we did not exclude those with major depressive disorders, as our sample primarily consists of healthy older adults living independently. This study protocol was approved by the Human Research Protections Program of the University of California, San Diego, and written informed consent was obtained from all study participants.

In-person interviews by a trained professional were conducted between April 2018 and January 2020. The interviewer and other staff were trained to administer neuropsychological tests according to standardized procedures by a licensed neuropsychologist (EWT), who was continuously available to answer scoring questions for the duration of the study. A comprehensive battery of neuropsychological assessments was planned, but the follow-up neuropsychological assessments were modified due to the COVID-19 pandemic. The neuropsychological tests included in these analyses were limited to those that were suitable for remote assessment (via telephone or videoconference). Of the 71 individuals who had completed interviews and assessments at baseline, 55 individuals had follow-up assessments (46 assessments were recorded at ~12 months, 6 assessments at ~18 months, and 3 assessments at ~24 months) with a mean follow-up interval of 1.4 years (512.3, SD 84.5 days). Of those, 37 assessments had all included measures available. Participants who did not complete a follow-up assessment had either moved out of the facility, moved to a higher level of care (eg, a nursing facility), declined further participation, or died.

### Sociodemographic and Clinical Neuropsychological Measures

#### Overview

The sociodemographic data collected included age, sex, marital status, and racial or ethnic background (Tables 1 and 2). Trained staff also administered a neuropsychological battery [19] to assess different cognitive domains. Information on the psychometric properties of the battery constituents can be found in Section S1 in Multimedia Appendix 1.

**Table 1:** Demographic characteristics of the sample at baseline (N=71).

				Value		Percentage impaired
**Sociodemographics**						
	**Age (in years at the time of visit)**					N/A^a^
		Mean (SD)	83.3 (7.0)			
		Range	67-98			
	**Education (in years)**					N/A
		Mean (SD)	15.9 (2.3)			
		Range	12-20			
	Marital status (single), n (%)		45 (63)		N/A	
	Race (White), n (%)		65 (91)		N/A	
	Sex (female), n (%)		47 (66)		N/A	
**Clinical measures**						
	**MoCA^b^**					39.44
		Mean (SD)	23.8 (3.7)			
		Range	13-29			
	**HVLT^c^ total recall**					22.39
		Mean (SD)	21.3 (5.3)			
		Range	9-31			
	**HVLT delayed recall**					28.36
		Mean (SD)	6.2 (3.5)			
		Range	0-12			
	**HVLT retention**					31.34
		Mean (SD)	67.5 (31.8)			
		Range	0-122			
	**FAS^d^**					29.41
		Mean (SD)	35.3 (11.3)			
		Range	16-61			
	**Animals^e^**					30.88
		Mean (SD)	16.6 (5.4)			
		Range	8-32			
	Overall deficit				71.83	
	**Depression PHQ-9^f^**					N/A
		Mean (SD)	3.2 (3.8)			
		Range	0-15			

^a^N/A: not applicable.

^b^MoCA: Montreal Cognitive Assessment.

^c^HVLT: Hopkins Verbal Learning Test-Revised.

^d^F-A-S verbal fluency test.

^e^Verbal processing speed (animals).

^f^PHQ-9: 9-item Physical Health Questionnaire.

**Table 2:** Demographic characteristics of the sample at follow-up (N=55).

				Value		Percentage impaired
**Sociodemographics**						
	**Age (in years at the time of visit)**					N/A^a^
		Mean (SD)	84.1 (7.5)			
		Range	68-100			
	**Education (in years)**					N/A
		Mean (SD)	16.3 (2.2)			
		Range	12-20			
	Marital status (single), n (%)		42 (93)		N/A	
	Race (White), n (%)		40 (89)		N/A	
	Sex (female), n (%)		30 (67)		N/A	
**Clinical measures**						
	**MoCA^b^**					35.14
		Mean (SD)	24.3 (4.3)			
		Range	6-29			
	**HVLT^c^ total recall**					42.22
		Mean (SD)	19.6 (6.6)			
		Range	5-31			
	**HVLT delayed recall**					42.22
		Mean (SD)	5.7 (3.7)			
		Range	0-12			
	**HVLT retention**					42.22
		Mean (SD)	63.2 (34.1)			
		Range	0-100			
	**FAS^d^**					29.55
		Mean (SD)	35.0 (9.3)			
		Range	18-53			
	**Animals^e^**					43.18
		Mean (SD)	16.1 (5.0)			
		Range	5-27			
		Overall deficit			78.26	
	**Depression PHQ-9^f^**					N/A
		Mean (SD)	3.5 (4.0)			
		Range	0-17			

^a^Not applicable.

^b^MoCA: Montreal Cognitive Assessment.

^c^HVLT: Hopkins Verbal Learning Test-Revised.

^d^F-A-S verbal fluency test.

^e^Verbal processing speed (animals).

^f^PHQ 9: 9-item Physical Health Questionnaire.

#### The Montreal Cognitive Assessment

The Montreal Cognitive Assessment (MoCA) [20] is a brief cognitive screening test to identify and stage dementia and includes items measuring attention, working memory, orientation, and short-term memory.

#### Hopkins Verbal Learning Test-Revised

The Hopkins Verbal Learning Test-Revised (HVLT) [21,22] is a list-learning and memory test that includes total recall (ie, total recalled words over 3 learning trials), a delayed recall trial, and a recognition trial.

#### Delis-Kaplan Executive Function System Verbal Fluency

This subtest [23] includes tests of verbal processing speed requiring the participant to name as many animals as possible in 1 minute and to name as many words starting with the letters F, A, and S in 1 minute.

#### Deficits Scores

For HVLT and Verbal Fluency tests, normative data from respective test manuals were used to convert raw scores to age-corrected T-scores. Each of the T-scores was converted into binary deficit scores. Deficits were defined as scores <23 on the MoCA or T-scores <40 on the remaining tests (reflecting >1 SD below the mean).

#### Overall Deficit

A composite binary variable was constructed by combining the MoCA, HVLT, and Verbal Fluency tests (0=no deficit, 1=deficit on any test).

### Acoustic Features

This study used a standardized procedure for qualitative interview processing as outlined by previous research [24,25]. Acoustic files (digital speech standard) were obtained following testing and converted to .wav format using NCH Switch Audio Converter (NCH Software). The audio files included the interviewer’s speech; however, we note that although multiple staff members were involved in administering the battery of neuropsychological assessments, the interviews were conducted by the same person and included a common set of questions, see Section S2 in Multimedia Appendix 1 for details. To reduce the number of parameters, we used the feature set “eGeMAPSv02,” which is based upon the Geneva minimalistic acoustic parameter set for voice research and affective computing, which identifies a basic set of acoustic features commonly used in clinical speech analysis [26]. A total of 88 Geneva minimalistic acoustic parameter set acoustic features are identified in the Python openSMILE library, which has been previously validated for this purpose [27-29]. These features were then extracted from the entirety of the interview using openSMILE audio processing software (audEERING GmbH). Notable among these were features of spectral slope (alphaRatioUV, slope UV 500-1500 HZ) and balance or shape or flux (Mel-frequency cepstral coefficient). F0 in the context of acoustic features generally refers to the fundamental frequency or the lowest resonating frequency produced by the vocal tract, while F1, F2, and F3 refer to higher resonating frequencies seen as successive peaks in the frequency spectrum. These are also referred to as formants. More details on the acoustic features can be found in Section S3 in Multimedia Appendix 1.

### Psycholinguistic Features

Linguistic features were extracted from the transcripts for each sample, drawing from established methodologies such as those detailed in Yamada et al [30] and other relevant literature [31]. In addition, we also explored the use of other higher-level psycholinguistic elements to assess language attributes, like readability, coherence, and references to forgetfulness and recollection. The interviews were transcribed using third-party services and were subsequently reviewed and verified manually.

In total, 86 distinct features were extracted to represent various linguistic traits: text statistics that denote parts of speech, vocabulary richness, grammatical complexity gauged by the nesting of phrases (Yngve depth) [32], verbal overlap between sentences (measured through cosine similarity [33,34]), sentiment analysis, readability metrics [35], and other features based on sentence embeddings generated by advanced language models such as the Bidirectional Encoder Representations from Transformers (BERT) [36]. Detailed interpretations of these features can be found in Table S1 in Multimedia Appendix 1.

### Data Analysis

After incorporating sociodemographic variables, like age, gender, education, race (categorized as White, Black, or Other), and ethnicity (distinguished as Hispanic or non-Hispanic), the total feature set for classification encompassed 178 distinct features.

Predicting baseline and future cognitive deficits often uses traditional regression techniques such as linear regression analysis or mixed linear modeling. However, due to observed limitations in model fit with our data set, a classification approach was deemed more appropriate. Target variables were dichotomized using cutoffs based on literature recommendations. This adaptation not only improved model accuracy but also aligned with clinical standards, specifically diagnostic cutoffs like scores below 23 on the MoCA [37] or T-scores 1 SD below the mean [38].

Owing to the limited understanding of the audio feature space in the context of psychiatry and the sparse body of work on psycho-linguistic features, 6 diverse ML models were separately explored to determine their performance. This was done in part to characterize the problem space: a high-performing support vector machine model or k-nearest neighbor might suggest distinct regions or clusters in the feature space, which could be pursued for future investigation. The models were retrained for the follow-up assignments, in order to identify potential lead-lag effects. To address the risk of overfitting, the feature space was reduced to the top 10 using the Gini impurity index [39,40] and the performance results (ie, *F*_1_-score, sensitivity, and specificity) were reported using leave-one-out cross-validation.

The following ML algorithms were used in the investigation: k-nearest neighbor, support vector machine, random forest, neural network, and naïve Bayes classifiers. Importantly, gender was considered alongside acoustic features in the ML analysis due to its potential impact on various acoustic properties, including pitch, which could represent a confounding variable. The hyperparameters applied across these models are exhaustively detailed in Table S2 in Multimedia Appendix 1.

The relative contribution of the features across domains was assessed by calculating the correlation between sociodemographic, acoustic, and psycholinguistic features with both raw scores and dichotomized deficits on neuropsychological subscales. Subsequently, the maximum correlation value for each feature with either the raw scores or deficit scores was determined, and the features were prioritized based on these values. The principal 25 features were then depicted in a heat map to facilitate the examination of their interrelationships. In addition, biclustering techniques [41] were used to delve deeper into the feature interdependencies. The emergent correlation matrix was visualized through a heat map, providing a comprehensive synopsis of the variable associations.

### Ethical Considerations

The study procedures and data analyses were reviewed and approved by the University of California San Diego Human Research Protections Program (Institutional Review Board #170466). The participants provided written informed consent to participate in this study. All data were anonymized, by use of a manual review of each transcript to remove any proper names, addresses, or potentially identifying information. All participants whose data are presented here provided additional consent to the use of information extracted from audio recordings for research purposes. Participants were compensated US $75 for participation in the study, which included the clinical and neurocognitive tests and qualitative interviews detailed in this manuscript.

## Results

### Sample Characteristics

The sample’s age ranged from 67 to 98 years at the initial visit, with a mean age of 83.3 (SD 7.0) years (Table 1). Most participants were single (n=45, 63%), White (n=65, 91%), and female (n=47, 66%), and had a high level of education (mean 15.9, SD 2.3 years). Cognitive functioning varied among participants, as indicated by MoCA scores ranging from 13 to 29. Depression scores were low (mean 9-item Patient Health Questionnaire score of 3.2, SD 3.8), rendering the sample inadequate for investigating the intersection of cognitive impairment with depression. The average time to the follow-up visit was 1.4 years (mean 512.3, SD 84.5 days) postbaseline (refer to Table 2). Attrition was primarily due to a lack of interest (n=27, 21%), transfer to more intensive care (n=11, 9%), participant deaths (n=11, 9%), and medical issues (n=3, 2%). A minority (n=3, 2%) reportedly withdrew due to cognitive decline. The reasons for the decrease in participation were not further analyzed.

### Classification Performance Using Acoustic and Psycholinguistic Features

The initial assessment focused on the effectiveness of various feature groups in distinguishing individuals with cognitive impairment from those without at baseline. To this end, *F*_1_-scores were used due to their balanced consideration of precision and recall, which is critical in the context of an uneven class distribution. Table 3 shows *F*_1_ performance scores, sensitivity, and specificity for various feature groups. Assuming a threshold of 0.75 for *F*_1_-scores (see Section S4, Multimedia Appendix 1 for the choice), acoustic and psycholinguistic features were acceptable and generally higher than *F*_1_-scores of sociodemographic features. Given the limited performance, sociodemographic features (gender, age, race, years of education) were best at predicting HVLT total recall (or learning) deficits compared to other deficits. Acoustic features were the best predictors of animal naming and overall deficits, and the psycholinguistic features performed best for HVLT total recall. The performance for MoCA and HVLT retention was comparable for both acoustic (*F*_1_ of 0.76 and 0.71, respectively) and psycholinguistic feature (*F*_1_ of 0.78 and 0.70, respectively) sets. Acoustic features performed better than psycholinguistic features in HVLT delayed recall (*F*_1_ of 0.72 vs 0.67), animal naming (*F*_1_ of 0.80 vs 0.70), and letter fluency (*F*_1_ of 0.78 vs 0.73), while psycholinguistic features performed better than acoustic in HVLT total recall (learning; *F*_1_ of 0.82 vs 0.77). As expected, the combined set (acoustic, linguistic, and sociodemographic features) performed the best among all individual feature sets. Gender did not play a factor among the top 10 features for any target in the baseline or the follow-up visit.

**Table 3:** Performance of features (by categories) for classification of deficits in baseline visit using top 10 features.

Target	Sample size	Sociodemographic top model, *F*_1_ (sensitivity, specificity)	Acoustic^a^ top model, *F*_1_ (sensitivity, specificity)	Psycholinguistic top model, *F*_1_ (sensitivity, specificity)	Combined^b^ top model, *F*_1_ (sensitivity, specificity)
MoCA^c^	71	ANN^d^ logistic, 0.62 (0.39, 0.79)	NB^e^, 0.76 (0.71, 0.79)	ANN logistic, 0.78 (0.64, 0.88)	NB, 0.80 (0.79, 0.81)
HVLT^f^ total recall T deficit score	67	NB, 0.74 (0.27, 0.90)	NB, 0.77 (0.67, 0.79)	NB, 0.82 (0.60, 0.88)	NB, 0.85 (0.73, 0.88)
HVLT delayed recall T deficit score	67	NB, 0.60 (0.05, 0.92)	ANN ReLu, 0.72 (0.42, 0.85)	ANN ReLu, 0.67 (0.32, 0.83)	NB, 0.74 (0.74, 0.73)
HVLT retention T deficit score	67	NB, 0.64 (0.14, 0.98)	NB, 0.71 (0.62, 0.74)	ANN tanh, 0.70 (0.48, 0.80)	NB, 0.73 (0.90, 0.63)
Animals T deficit score^g^	68	ANN tanh, 0.61 (0.29, 0.77)	NB, 0.80 (0.81, 0.79)	NB, 0.70 (0.57, 0.74)	NB, 0.80 (0.71, 0.83)
FAS T deficit score^h^	68	Random forest, 0.65 (0.30, 0.81)	NB, 0.78 (0.85, 0.73)	SVM^i^, 0.73 (0.40, 0.90)	NB, 0.76 (0.75, 0.75)
Overall deficit score	71	ANN ReLu, 0.69 (0.76, 0.50)	NB, 0.81 (0.84, 0.70)	NB, 0.81 (0.76, 0.90)	NB, 0.86 (0.84, 0.90)

^a^Incudes gender as a feature.

^b^Combined: acoustic, psycholinguistic, and sociodemographic features.

^c^MoCA: Montreal Cognitive Assessment.

^d^ANN: artificial neural network.

^e^NB: naïve Bayes.

^f^HVLT: Hopkins Verbal Learning Test.

^g^Animals T-score (D-KEFS Norms).

^h^FAS total T-score (D-KEFS Norms).

^i^SVM: support vector machine.

Using baseline features to determine cognitive status at subsequent follow-up visits (Table 4), it was observed that the predictive capacity of these features remained consistent across tests (for instance, an *F*_1_-score of 0.87 was noted for MoCA using all features). This observation was in line with the initial analysis, where psycholinguistic features outperformed acoustic features, which in turn were more predictive than sociodemographic variables. There was also an improvement in the performance of sociodemographic features (*F*_1_-scores; Table 4) and sensitivity (of >0.5 in most cases, Table 4), but they continued to underperform compared with the acoustic and psycholinguistic features. MoCA (*F*_1_=0.87, sensitivity=0.85, specificity=0.88) and letter fluency (*F*_1_=0.91, sensitivity=1.00, specificity=0.87) were the best predicted cognitive tests for a year in the future. Among the ML models used, the naïve Bayes classifier generally performed better than other models for most classification targets (Table S3, Multimedia Appendix 1). In addition, we performed a 10-fold cross-validation; the results aligned closely with leave-one-out cross-validation for best-performing models and are reported in Table S4 in Multimedia Appendix 1.

**Table 4:** Performance of features (by categories) for classification of deficits in follow-up visits using top 10 features.

Target	Sample Size	Sociodemographic top model, *F*_1_ (sensitivity, specificity)	Acoustic^a^ top model, *F*_1_ (sensitivity, specificity)	Psycholinguistic top model, *F*_1_ (sensitivity, specificity)	Combined^b^ top model, *F*_1_ (sensitivity, specificity)
MoCA^c^	37	ANN^d^ logistic, 0.72 (0.54, 0.83)	NB^e^, 0.79 (0.92, 0.71)	NB, 0.87 (0.85, 0.88)	ANN tanh, 0.87 (0.85, 0.88)
HVLT^f^ total recall T deficit score	45	Random forest, 0.64 (0.58, 0.69)	NB, 0.71 (0.74, 0.69)	NB, 0.69 (0.63, 0.73)	ANN ReLu, 0.76 (0.79, 0.73)
HVLT delayed recall T deficit score	45	NB, 0.71 (0.58, 0.81)	NB, 0.69 (0.68, 0.69)	NB, 0.82 (0.79, 0.85)	NB, 0.87 (0.84, 0.88)
HVLT retention T deficit score	45	ANN logistic, 0.71 (0.63, 0.77)	NB, 0.69 (0.68, 0.69)	NB, 0.76 (0.68, 0.81)	NB, 0.82 (0.84, 0.81)
Animals T deficit score^g^	44	Random forest, 0.66 (0.58, 0.72)	NB, 0.77 (0.84, 0.72)	NB, 0.70 (0.63, 0.76)	NB, 0.82 (0.79, 0.84)
FAS T deficit score^h^	44	ANN tanh, 0.70 (0.46, 0.81)	NB, 0.84 (0.77, 0.87)	NB, 0.89 (1.00, 0.84)	NB, 0.91 (1.00, 0.87)
Overall deficit score	46	ANN tanh, 0.81 (0.94, 0.40)	Random Forest, 0.78 (0.86, 0.50)	ANN Logistic, 0.80 (0.89, 0.50)	NB, 0.89 (0.92, 0.80)

^a^Incudes gender as feature.

^b^Combined: acoustic, psycholinguistic, and sociodemographic features.

^c^MoCA: Montreal Cognitive Assessment.

^d^ANN: artificial neural network.

^e^NB: naïve Bayes.

^f^HVLT: Hopkins Verbal Learning Test.

^g^Animals T-Score (D-KEFS Norms).

^h^FAS Total T-Score (D-KEFS Norms).

### Ranking and Proportion of Acoustic and Psycholinguistic Features and Demographics

Overall, acoustic features performed best in classifying animal fluency, letter fluency, and HVLT total recall (learning), while psycholinguistic features performed best in classifying MoCA and HVLT retention (Table 5). The prominence of acoustic features in deficit prediction prompted us to establish age correlates of acoustic features (Table S5, Multimedia Appendix 1). Few sociodemographic features were present in the top 10 features. Among acoustic features, the most predictive included ones corresponding to nonverbal vocalizations of energy and its distribution across frequencies (formants) in absolute ranges of 500-1500 Hz (slopeUV), as well as in terms of individual physiology (Mel-frequency cepstral coefficient), and jitter. Variation in loudness was also an important acoustic feature in identifying cognitive deficits. Among the psycholinguistic features, the amount of speech produced (number of transcribed characters and words, number of utterances) along with vocabulary richness (vocabulary type token ratio, interview readability), proportion of nouns (noun to verb ratio, verb frequency, pronoun, and particle frequency), repetition (cosine similarity), and sentiment were important correlates of cognitive deficits.

**Table 5:** Contribution profile of deficits based on top contributing features at the baseline visit. MoCA^a^ scores are dominated by psycholinguistic predictors. HVLT^b^ total and delayed recall, animal naming, and FAS^c^ are all predominantly determined by acoustic features. Sociodemographic features play a minor role in HVLT total recall and the overall deficit.

Cognitive assessment target	Acoustic (%)	Psycholinguistic (%)	Sociodemographic (%)
MOCA	10	90	0
HVLT total recall	80	10	10
HVLT delayed recall	70	30	0
HVLT retention	40	60	0
Animals	70	30	0
FAS	70	30	0
Overall deficit	20	70	10

^a^MoCA: Montreal Cognitive Assessment.

^b^HVLT: Hopkins Verbal Learning Test-Revised.

^c^F-A-S verbal fluency test.

### Clustering and Heat Map of Features and Targets

The raw scores of HVLT and MoCA tasks were clustered in a subtree, while all deficits were in a separate subtree. The clustering suggested that animal naming deficits very closely resembled the derived overall deficit.

**Figure 1:**
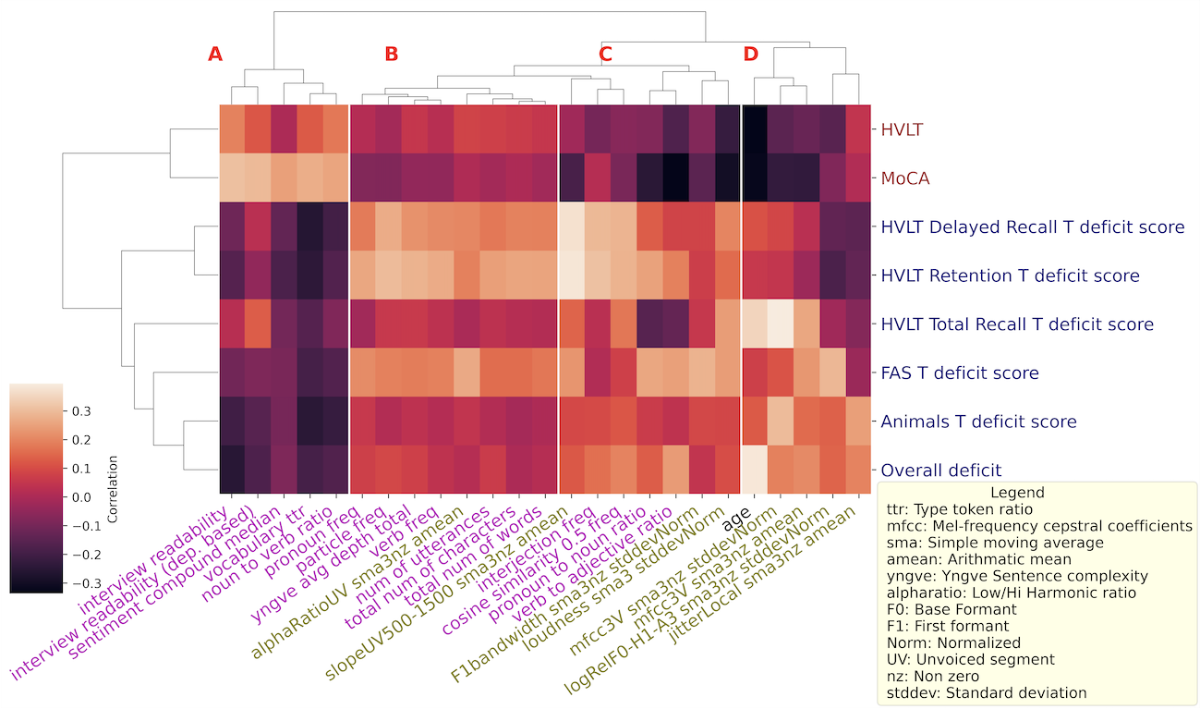
Heat map and results of biclustering of top 25 features by correlation. Along the x-axis, the acoustic features are in green, psycholinguistic features in pink, and sociodemographic are in black. Along the y-axis, the task scores are in brown and the deficits in blue. Four clusters of features are evident, (A) and (B) being predominantly psycholinguistic and (D) predominantly acoustic. (A) Lexical richness and positive sentiment: the group of features reflect vocabulary richness (interview readability, vocabulary type token ratio, noun to verb ratio) and sentiment that relates positively with the cognition scores and negatively with deficits. (B) Reversal of concreteness and greater speech production: comprises mostly of psycholinguistic features that suggest replacement of nouns by descriptive phrases (pronoun frequency, particle frequency, verb frequency, number of utterances, number of transcribed characters and words, yngve depth) but one acoustic feature is also included (mean alpha ratio UV simple moving average). These features correlate positively with some deficits and negatively with others and show a certain homogeneity, the cluster associates with increased language output. (C) Speech fragmentation: the cluster seems to encode speech fragmentation through interjection (interjection frequency), repetition (cosine similarity) and loudness variation (norm of loudness standard deviation), and audio features for spectral balance in absolute frequency terms (mean slope UV 500-1500 Hz and F1 bandwidth) correlating negatively with cognition scores and positively with the deficits. (D) Nonverbal vocalizations: acoustic features representing spectral balance or shape or dynamics in individual vocal tract and glottis physiology (mean and SD of mel-frequency cepstral coefficients, jitter) and age, that corelates negatively with the cognition scores but positively with deficits and can encode nonverbal emotions. HVLT: Hopkins Verbal Learning Test-Revised; MoCA: Montreal Cognitive Assessment.

Our analysis indicated that the set of top contributing features across all targets was clustered into 4 subtrees. The cluster labeled A (Figure 1) was comprised of psycholinguistic features representing vocabulary richness and other metrics of vocabulary use including type token ratio, noun-verb ratio, interview readability, and sentiment. These features correlated positively with task scores and negatively with deficits. The cluster labeled B in Figure 1 predominantly contains psycholinguistic features representing increased speech production. Included in this cluster were features corresponding to particle, verb, and pronoun use, increased utterances, transcribed characters, and words. The cluster labeled C also predominantly includes psycholinguistic properties features and contains features that represent speech fragmentation. This includes features that punctuate language (interjections) and repetition (cosine similarity), and also present were acoustic features representing variation in loudness (slopeUV500-1500, loudness) or the control of glottis closure. These features correlated positively with deficits and negatively with raw cognitive scores. In contrast to clusters A, B, and C, the cluster labeled D was comprised of almost all acoustic features representing spectral balance and tilt.

## Discussion

### Principal Findings

A manual review of the cognitive measures of participants along the timeline revealed abrupt changes that were not always monotonic (strictly decreasing) as might be expected. In fact, MoCA has been shown to significantly improve in the second administration [42]. These effects combined with the noise in the testing, as well as the within-subject fluctuations, make regression over short intervals difficult. Furthermore, individuals transferred to higher care facilities, who may have shown greater declines, were not included in this analysis. The research and medical team agreed that modeling individual decline over 1.4 years might not be feasible. Posing it as a classification problem by dichotomization, however, allowed us to model age-related changes at a very broad level with a modest degree of success.

This work is among the first to evaluate both acoustic and psycholinguistic features in a longitudinal study of cognitive performance in an older adult sample. We found that both acoustic and psycholinguistic features provided a reasonably strong classification of overall cognitive impairment, verbal memory, and verbal fluency tasks at both baseline and follow-up assessments. While using psycholinguistic features and sociodemographic features alone, the classification of cognitive impairment was similar to previous approaches [13]. Combining the acoustic and psycholinguistic features, however, enhanced the classification performance. We found that baseline acoustic and psycholinguistic features predicted future performance on the same cognitive tasks at a mean follow-up of 1.4 years. Comparing the relative accuracy of acoustic versus psycholinguistic features, there was a slight disadvantage of acoustic features compared to psycholinguistic features using the *F*_1_-score metric in the follow-up. Features clustered by type and varied across cognitive tasks, suggesting that different features may be useful for detecting different aspects of cognitive impairment. Since the actual performance scores (*F*_1_-scores) were in the 0.7-0.8 range, separation boundaries were expected to be fuzzy. Overall, our findings add to the growing body of literature indicating that linguistic and acoustic analysis of speech samples may aid in the detection of cognitive deficits in aging.

Our findings are consistent with prior research that has investigated psycholinguistic markers of cognitive aging. A change, generally a reduction, in noun production, has long been identified as an early indicator of cognitive decline by several studies [43-45]. Nouns and verbs are the most common categories in the English language, and their use often declines in early dementia [46]. Word-finding difficulties foreshadow progressive aphasias and other degenerative dementias [47,48]. In our heat map–based analysis of the baseline data, we found that lexical richness related negatively to deficits and positively to cognitive performance. The reduced access to nouns due to a decline in semantic networks may initially result in longer phrases that describe the nouns, thus resulting in increased language production in social settings [49,50]. This may also be related to as “reversal of concreteness effect” [51-53]. This phenomenon is reflected in the features that represent an increase in the frequency of pronouns, particles, and verbs (eg, the inability to recall the word “fork” could result in saying “that thing you eat with,” such that a noun is replaced by several other parts of speech) and increase in utterances, words and characters, and even coverbal gestures [54]. Grammatically, this would cause an increase in the depth of the parse tree, the Yngve depth [32], as a single word gets replaced by a phrase. Written grammatical complexity, however, decreases over longer timeframes, as suggested by a study spanning the lifetime of a novelist with Alzheimer disease [55]. Notably, we did not predict decline per se but rather future cognitive ability. Eyigoz et al [13] demonstrated the predictive value of linguistic features over a 7-year period in reference to conversion to Alzheimer disease. It is possible that our mean follow-up period of 1.4 years was too short to result in a significant number of individuals becoming impaired.

Our study adds to the comparatively smaller body of literature on acoustic features in cognitive aging. Exploring these features is important given that not all are detectable with the human ear, despite some evidence that they may covary with age and cognitive ability. Acoustic features relating to the Mel-frequency cepstral coefficient may have embedded within them artifacts of age-related decline [56,57] leading to the poorer representation of nonverbal vocalizations, possibly through the expression of emotions through glottis control, for example, laughter or giggle [58]. Some top predicting acoustic features were also associated with variation in loudness [59] or the glottis closing slowly or insufficiently due to aging [60]. Such nonverbal vocalizations are not correlated with grammar [61,62], so they are undetected by NLP extraction tools. Some, such as laughter or giggle, are produced in glottal or subglottal structures spontaneously and are in the annotation category, while others such as breathing, correlate with pauses in prosodic hierarchy [63]. Aging is also reflected in jitter [64] due to vocal fold atrophy [65]. Overall, it was notable that our best models combined acoustic and psycholinguistic features, and that our heat map suggested that different cognitive domains were predicted by unique feature sets that clustered with acoustic features or psycholinguistic. As such, future research should evaluate the dynamics of within-person change in acoustic and linguistic features as they may predict changes in cognitive performance in a range of cognitive tasks over time.

It is possible that the decline manifests itself in fits and bursts due to the noise in the measurement of cognitive tasks and within-individual fluctuations: the changes are small over the interval of 1.4 years compared to the overall value. Furthermore, individuals who were transferred to a higher level of care may have a greater decline but could not be included in this analysis. Hence, our data does not allow for the prediction of various cognitive scores on an individual basis. However, if the scores are dichotomized and aggregated, the predictions about the cognitive status change can achieve performance that is suitable for screening purposes. Future studies with larger samples and longer periods of follow-up would allow for finer-grained prediction of cognitive decline in specific domains.

One study [66] has documented extensively the performances of a variety of BERT-based transformers on this task. We found that most of these models under-performed our combined models, and only one approached a comparable performance (BERT_Large_-LR; *F*_1_-scores of 87%). This, in our opinion, can be attributed to our rich feature set that included acoustic features that potentially convey important information about the physical health of the participants. The use of large language models (such as ChatGPT) was avoided due to two concerns: (1) the models are criticized for opaqueness to feature interpretation, which was our focus, and (2) patient privacy concerns. At the time of our research, the available generative large language models did not meet our specific needs in terms of local deployment capabilities and data sensitivity [67] (ie, ChatGPT and Claude could not be run locally). We are excited to note that with the introduction of open-source models, such as Llama 3, which offers robust performance while being feasible for local deployment, we plan to bridge this gap.

### Limitations

There are some limitations in this study. The sample was relatively homogenous, with high socioeconomic status, primarily White, and highly educated, relative to the broader older adult population. The acoustic recordings were roughly 1.5-2 hours in length, and thus the minimum length of speech data needed for these analyses is unknown. The current interview length may not be practical for clinical deployment. These analyses include acoustic features extracted from the entire interview; the fraction of the interviewer’s speech was small and there were fixed questions. Although the same interviewer conducted the interview with the same set of prompts, it is reasonable to expect that some bias was introduced in the acoustic features of the interviewee resulting in some loss of performance of ML models. Other limitations include that drop out was significant and longitudinal prediction may be affected by this; as well, there was a variable follow-up duration. We also had planned a broader neuropsychological battery with nonverbal domains, but the transfer to a remote assessment paradigm due to the COVID-19 pandemic prevented this. In addition to impacting the shift to remote assessments, the COVID-19 pandemic may have impacted aspects of daily life that may influence cognitive ability (eg, access to physical activity), therefore, the findings should be interpreted in this light.

Finally, while we did perform leave-one-out cross-validation, we lacked an independent sample with which to validate these features or establish confidence intervals. Model fits were evaluated based solely on *F*_1_-score comparisons. Constrained by the mentioned limitations, the study should be considered exploratory.

### Conclusions

Despite these limitations, our study raised questions that warrant future investigation. We were unable to ascertain if the features most indicative of cognitive impairments are also predictive of those likely to experience decline in the near future. Furthermore, the mapping between the acoustic features and cognitive domains remains unclear. The value of adding acoustic and psycholinguistic features to assessments and the duration of speech needed to discriminate cognitive impairments could be contrasted to the relative importance beyond that associated with cognitive screening data. Although our semistructured interview was broad, it would be useful to understand both the impact of the nature of the prompts and topics covered, as well as the minimum duration of speech sample required, to achieve the desired accuracy in predicting cognitive deficits. The generalizability of acoustic and psycholinguistic features, especially beyond English-speaking and primarily well-educated White groups, requires confirmation across different languages and racial or ethnic backgrounds. Overall, our results suggest a potential role for acoustic and psycholinguistic data in cognitive assessment; the next step is to determine the timing and connection to brain changes that occur with pathological aging processes such as in dementia.

## Appendix

Multimedia Appendix 1 Supplementary Material.

## Abbreviations

BERT: Bidirectional Encoder Representations from Transformers
HVTL: Hopkins Verbal Learning Test-Revised
MCI: mild cognitive impairment
ML: machine learning
MoCA: Montreal Cognitive Assessment
NLP: natural language processing

## Data Availability

The datasets generated during or analyzed during this study are not publicly available due to privacy concerns, including Health Insurance Portability and Accountability Act regulations, but the code for generating custom features of machine learning model parameters is available from the corresponding author upon reasonable request.
