# Supplementary material for: Investigating Acoustic and Psycholinguistic Predictors of Cognitive Impairment in Older Adults: Modeling Study
Source: JMIR Aging. 2024 Sep 16;7:e54655. doi: 10.2196/54655 (PMC11443203; doi:10.2196/54655)
Supplement: Multimedia Appendix 1 [file aging_v7i1e54655_app1.docx]

**Supplementary Material**

**Section S1: Psychometric properties of constituents of neuropsychological battery**

The psychometric properties of MoCA are documented across the adult lifespan (18-70 years) [1], which documents the MoCA Total Score test-retest reliability (across versions 7.1 , 7.2 and 7.3) as good to excellent (ICC between 0.64 and 0.82 ).The Hopkins Verbal Learning Test-Revised (HVLT-R) has a moderate test-retest reliability of 0.38 to 0.65, and high reliability (Cronbach’s α 0.94) [2]. An update on reliability and validity of Delis-Kaplan Executive Function System has been compiled from scientific journals for various study populations and can be found here [3].

**Section S2: Qualitative questions in the semi-structured interviews that was analyzed in the current project.**

The interview had four main sections: relationships with friends and family, loneliness, perceptions of aging, and technology. The prompts are listed below:

1. **Relationships with Family and Friendship**

- Do you have important relationships in your life? Please describe them.
- What makes those relationships meaningful to you?
- Do you feel that there are people in your life who fully understand you?
- How often do you spend time with or connect (via phone, email, or social media) with others?
- Do you feel you are part of a larger community? Please explain.
- When you are feeling disconnected or isolated what do you do?

1. **Loneliness**

- If you could design a perfect day, what would you be doing, who would you be with, how would you feel?
- Do you ever feel lonely, and if so, how often?

If participant responds that they **do** feel lonely:

- What does loneliness feel like to you? What is your general mood during that time?
- Is this something new or has this been an experience throughout your life? Please explain.
- When you are feeling lonely, what do you do? Does that help you?
- How can others help reduce your loneliness?

If participant responds that they **do not** feel lonely:

- Why do you think others may feel lonely?
- What do you think loneliness feels like to them?
- How might aging play a role in loneliness? (Do you think feeling lonely affects aging?)
- What do you think people can do to not feel lonely anymore?

1. **Perceptions of Aging**

**Successful Aging**

- How do you define successful aging?
- What is important to aging successfully?
- Would you consider yourself to be aging successfully? Why or why not?
- Do you have any suggestions on how to age well or recommendations for specific interventions to promote healthy aging?

**Meaning and Purpose in Life**

- Thinking about your life, what do you live for? (Has this changed over time?)
- What has brought you the greatest meaning in your life?
- What would be your life mission statement?

**Wisdom**

- How would you define wisdom?
- Do you consider yourself a wise person?
- How have your life experiences influenced your personal wisdom?
- What are the main characteristics of someone who is wise?

1. **Technology and Successful Aging**

- What do you think about technology? What type of technology do you use, if any?
- How do you think that wearable sensors, smartphone apps, in-home technology, or robots could help support older adults in being more independent?
- Do you think that these devices could be used to reduce loneliness? Could they be used to prevent falls?
- If you could develop technology to improve your life in any way, what would you develop?

**Section S3: Description of Top Acoustic Features.**

The Geneva Minimalistic Acoustic Parameter Set (GeMAPS), as the name suggests, is a minimal set of voice acoustic features that are deemed suitable for both voice (trait) and mood (state) related research. The effort was motivated by the proliferation of such features over the decades, and non-standard extraction methods. The feature set was arrived at using several INTERSPEECH challenges, with an aim to minimize size and maximize performance. The feature-set is standardized, but it is evolving based on new findings, indicated by the version number.

Features for individual targets were ranked using GINI impurity index**.** The GINI index or Gini impurity represents the probability (0 to 1) that the feature is wrongly classified (0 = “pure”, 0.5 = equal distribution across all classifications, 1 = random distribution across classes) [4, 5].

The Acoustic features are described in detail in [6] and [7]. Based on the analyses

| **Top Audio Features** | **Type** |
| --- | --- |
| alphaRatioUV_sma3nz_amean | Spectral balance |
| slopeUV-500-1500_sma3nz_amean | Spectral balance |
| F1bandwidth_sma3nz_stddevNorm | Formant Bandwidth |
| loudness_sma3_stddevNorm | Variation in loudness |
| mfcc3V_sma3nz_stddevNorm | Spectral balance |
| mfcc3V_sma3nz_amean | Spectral balance |
| logRelF0-H1-A3_sma3nz_stddevNorm | Creekiness/ Breathiness |
| jitterLocal_sma3nz_amean | Breathiness |

- **Spectral Balance:** These parameters are influenced by laryngeal and supra-laryngeal movements and are related to perceived voice quality and non-verbal vocalizations identified for communicating emotions [7, 8].
- **Formant Bandwidth**: These features indicate vowel intelligibility between the healthy and dysphonic speakers [9, 10].
- **Variation in Loudness:** These features reflect changes in voice amplitude possibly due to glottis closure or sporadic sound production.
- **Creakiness: These features reflect** a complex pattern of vibrations in the vocal folds sounding like two hard surfaces rubbing against one another [11].
- **Breathiness:** These features reflect that the sound of air is apparent or impression of glottal air leakage and turbulence noise during phonation [11, 12].

**Table S1: Description of top Linguistic Features** [13].

The description of features has also been described in a previous publication [14].

| **Top NLP Features** | **Type** |
| --- | --- |
| interview_readability | Vocabulary Richness |
| interview_readab_dep_based | Vocabulary Richness (alternative sentence tokenization) |
| sentiment_compound_median | Sentiment |
| vocabulary_ttr | Vocabulary Richness |
| noun_to_verb_ratio | Part-of-speech |
| pronoun_freq | Part-of-speech |
| particle_freq | Part-of-speech |
| yngve_avg_depth_total | Syntactic complexity |
| Verb_freq | Part-of-speech |
| Num_of_utterances | Amount of speech |
| total_num_of_characters | Amount of speech |
| total_num_of_words | Amount of speech |
| interjection_freq | Sentiment/ Filled pause |
| cosine_similarity_0.5_freq | Sentence similarity |
| pronoun_to_noun_ratio | Part-of-speech |
| verb_to_adjective_ratio | Part-of-speech |

**Preprocessing**

For preprocessing of the transcript data, we performed sentence tokenization, word tokenization, and part-of-speech tagging on the transcribed text by using the Natural Language Toolkit (NLTK) [15] and spaCy [16] Python libraries. We also performed lemmatization using Wordnet lemmatizer [17] available on NLTK.

**Linguistic features**

- Vocabulary Richness

The features related to vocabulary richness measure lexical diversity. These features have been linked to decline in patients with Alzheimer’s disease [18, 19] and patients with mild cognitive impairment [20].

There are three types of vocabulary richness features: type-token ratio (TTR), Brunét’s index (BI), and Honoré’s statistic (HS) [21]. TTR compares the total distinct word types (U) to the total word count (V) as $TTR=U/V$. Using the same U and V, BI is defined as $BI=V^{U^{-0.165}}$. Unlike other measures related to vocabulary richness, for this measure, the lexical richness becomes greater as BI becomes smaller. HS gives particular importance to unique vocabulary items used only once, also known as hapax legomena $V_{uni}$. HS is defined as $HS=100logV/(1-V_{uni}/U)$.

- Part-of-speech

Frequency and proportion of part-of-speech have been used as linguistic features to reflect cognitive functioning. For example, pronoun frequency has been used for quantifying difficulties with word-finding and word-retrieving in patients with Alzheimer’s disease and mild cognitive impairment. Previous studies on Alzheimer’s patients’ speech reported increased proportion of pronouns [18, 21-23], adjective [21, 23] and verbs [21, 23] and reduced proportion of nouns [21, 23]. We used frequency and proportion of the following part-of-speech tags as linguistic features: nouns, verbs, adjectives, pronouns, adverbs, conjunctions, particles, and interjections. For the proportion variables, we normalized the frequency by the total number of words. In addition, we used the proportion of nouns to verbs, pronouns to nouns, nouns to adjectives, and verbs to adjectives as features.

- Filled pause

Frequency and proportion of filled pauses have been used for measuring dysfluency in speech and quantifying language dysfunctions related to impaired lexical access, syntactic difficulties, discourse planning deficits [24]. The number of filled pauses have been reported to increase in case of Alzheimer’s disease [25], aphasia [26] and depression [27].

- Syntactic complexity

Features related to syntactic complexity have been used for quantifying syntactic impairments as well as memory and semantic difficulties [18, 28]. Several studies have found a decrease in the syntactic complexity of language in case of Alzheimer’s disease [19, 29, 30] and aphasia [26]. Features related to syntactic complexity include length metrics and parse tree metrics. For the length metrics, we used total number of sentences, words, and characters as well as their statistics across sentences including mean, median, minimum, maximum, standard deviation. Parse tree metrics include statistics of the Yngve depth (a measure of embeddedness) across sentences [31] and its total depth. Yngve depth was calculated by using Stanford CoreNLP package [32].

- Sentence similarity

Sentence similarity has been used for quantifying repetition or perseveration in the speech. Previous studies on patients with Alzheimer’s disease reported increased sentence similarities due to frequent repetition of specific words and phrases [33, 34].

One of the sentence similarity measures is calculated by using cosine similarity between sentences. We first converted sentences into term frequency–inverse document frequency (TF-IDF) vectors by using a bag-of-words model [35]. TF-IDF is a numerical statistic intended to reflect how important a word is to a document. By using TF-IDF vectors, we then calculated the cosine similarity between all pairs of sentences and used their statistics as well as the frequency and proportion of similar sentence pairs (>0.5). The statistics included mean, median, minimum, maximum, and standard deviation.

- Sentiment

Sentiment features are typically used for detecting changes in mental states. For example, depressed individuals were reported to tend to use more negative words and fewer positive words than non-depressed individuals [36, 37]. We used statistics of positive, negative, and neutral emotional scores for each sentence as well as a compound score for the document. The compound score ranges from -1 (most negative) to +1 (most positive). The scores were calculated by using VADER sentiment analyzer [38] with Python NLTK package [15].

- Readability

Readability is the ease with which a reader can understand a written text. This feature measures the readability index, which quantifies how easy or difficult it is for a person to read and understand the text based on the consensus of several different methodologies: Flesch reading ease formula, Flesch-Kincaid grade level, the Fog scale, the SMOG index, Automated readability index, Colemau-Liau index, Linsear write formula, and Dale-Chall readability score [39, 40].

**Table S2:** **Machine learning (ML) hyper-parameters.**

Due to number of models used and number of hyper-parameters involved, fine tuning of all hyper-parameters using fine gridding was not practical. Each ML model has its own set of hyperparameters. We attempted rough gridding, at times varying only orders of magnitude (e.g., gamma for SVM 0.01, 0.1, 1) to establish an operating scale. We did not attempt to refine performance by changing in small incremental steps. The choice was guided by essentially having reached a performance plateau. Owing to limited data set size, we consider any further such attempt having the same effect as overfitting. The study is hence exploratory.

| ***ML Model*** | ***Hyper parameters used*** |
| --- | --- |
| Naïve Bayes | Scipy implementation |
| ANN tanh | Scipy implementation, Number of hidden layers=1, Number of neurons in hidden layer = 200, solver=Adam |
| Random Forest | Number of Trees=8, Number of attributes for split=4, Limit depth=7, Don’t split subsets smaller than 2 |
| kNN | Number of neighjbors k = 9, Metric=Chebyshev, weight=distance |
| ANN Logistic | Scipy implementation, Number of hidden layers=1, Number of neurons in hidden layer = 200, solver=Adam |
| SVM RBF | Cost (C)=1, Numerical Tolerance = 0.001, Epsilon=0.10, g=auto |
| ANN ReLu | Scipy implementation, Number of hidden layers=1, Number of neurons in hidden layer = 200, solver=Adam |

ANN: Artificial Neural Network

kNN: k nearest neighbors

SVM: Support Vector Machine

RBF: Radial Basis Function

ReLu: Rectified Linear Unit

**Section S4:** **Technical Notes**

**F1 scores and cutoffs:** F1-score is a harmonic mean of precision and recall, and it ranges between 0 and 1, higher the better. There exists a strong argument and a general consensus on using F1-scores to evaluate relative merits of ML models [41]. However, there seems to be a considerable margin on a cutoff for acceptability of F1-scores as it strongly depends on the problem domain. For problems in the domains of physical sciences, the expectations are generally higher. ML metrics reported in medical domain [42] values for F1 scores range between 0.74 to 0.95. A large scale survey [43] report F1 score on a variety of medical applications ranging from FMRI imaging to NLP on clinical notes, where it ranges between 0.66- .96, 0.75 allowing for a good lower cutoff.

**LOOCV, F1 scores and confidence intervals:** We used an F1 score of 0.75 as our benchmark for satisfactory performance. The F1 scores we obtained were derived from Leave-One-Out cross validation (LOOCV) [44], ensuring that the results were neither overfitted nor influenced by a fortunate split between training and testing sets. However, upon further examination, we realized that with LOOCV, it's impossible to observe any variance in the results. This is because, once a model is trained using LOOCV, each example is precisely classified into one category. Since LOOCV utilizes all possible train-test set combinations, no variation in outcomes can be detected. Further, using bootstrapping (or any other such method) would severely underpower this study and would still not provide an unbiased estimator of variance [45].

**Advantages and disadvantages of LOOCV:** Using LOOCV has advantages. It has less bias because of the larger training set and repeated application always produces the same results (because it lacks randomness and uses all training and test sets) [45]. Only notable disadvantage is its time and computational intensiveness.

**Confidence intervals:** All features, sociodemographic, acoustic, and psycholinguistic, were extracted at baseline, only targets were changed. The sampling errors in features remained constant. Any performance differences should be attributed to the model fit. Leave One Out Cross Validation Analysis assures us they were not due to lucky portioning of test and train sets. Confidence interval analysis cannot be applied because each sample is classified exactly into one class, without any variance. Only new data can allow such analysis.

**Table S3: Top performing models for each feature set and cognitive domain target using leave one out cross- validation (LOO)** **for baseline value.**

| **Target** | **Socio-demographic** | | **Acoustic**^*^ | | **Psycholinguistic** | | **Combined**^**^ | |
| --- | --- | --- | --- | --- | --- | --- | --- | --- |
|  | **Model** | **F1** | **Model** | **F1** | **Model** | **F1** | **Best Model** | **F1** |
| MoCA^#^ | ANN Logistic | 0.62 | Naïve Bayes | 0.76 | ANN Logistic | 0.78 | Naïve Bayes | 0.80 |
| HVLT Total Recall T deficit score | Naïve Bayes | 0.74 | Naïve Bayes | 0.77 | Naïve Bayes | 0.82 | Naïve Bayes | 0.85 |
| HVLT Delayed Recall T deficit score | Naïve Bayes | 0.60 | ANN ReLu | 0.72 | ANN ReLu | 0.67 | Naïve Bayes | 0.74 |
| HVLT Retention T deficit score | Naïve Bayes | 0.64 | Naïve Bayes | 0.71 | ANN tanh | 0.70 | Naïve Bayes | 0.73 |
| Animals T deficit score^$^ | ANN tanh | 0.61 | Naïve Bayes | 0.80 | Naïve Bayes | 0.70 | Naïve Bayes | 0.80 |
| FAS T deficit score^^^ | Random Forest | 0.65 | Naïve Bayes | 0.78 | SVM | 0.73 | Naïve Bayes | 0.76 |
| Overall deficit score | ANN ReLu | 0.69 | Naïve Bayes | 0.81 | Naïve Bayes | 0.81 | Naïve Bayes | 0.86 |

^*^Incudes gender as feature

^**^Combined: acoustic, psycholinguistic, and socio-demographic features

^#^Montreal Cognitive Assessment

HVLT: Hopkins Verbal Learning Test

^$^Animals T-Score (D-KEFS Norms)

^^^FAS Total T-Score (D-KEFS Norms)

**Table S4: Top performing models for each feature set and cognitive domain target using K-10 fold cross- validation for baseline value.**

| **Target** | **Socio-demographic** | | **Acoustic**^*^ | | **Psycholinguistic** | | **Combined**^**^ | |
| --- | --- | --- | --- | --- | --- | --- | --- | --- |
|  | **Model** | **F1** | **Model** | **F1** | **Model** | **F1** | **Best Model** | **F1** |
| MoCA^#^ | ANN Logistics | 0.61 | *Naive Bayes* | 0.76 | ANN Logistic | 0.80 | ANN Logistic | 0.82 |
| HVLT Total Recall T deficit score | kNN | 0.74 | kNN | 0.78 | ANN tanh | 0.76 | *Naive Bayes* | 0.80 |
| HVLT Delayed Recall T deficit score | kNN | 0.60 | *Naive Bayes* | 0.70 | *Naive Bayes* | 0.70 | *Naive Bayes* | 0.73 |
| HVLT Retention T deficit score | *Naive Bayes* | 0.62 | *Naive Bayes* | 0.65 | ANN tanh | 0.70 | *Naive Bayes* | 0.68 |
| Animals T deficit score^$^ | ANN ReLu | 0.62 | *Naive Bayes* | 0.79 | *Naive Bayes* | 0.71 | *Naive Bayes* | 0.78 |
| FAS T deficit score^^^ | Random Forest | 0.67 | *Naive Bayes* | 0.75 | ANN Logistic | 0.73 | *Naive Bayes* | 0.77 |
| Overall deficit score | *Naive Bayes* | 0.72 | *Naive Bayes* | 0.78 | *Naive Bayes* | 0.87 | *Naive Bayes* | 0.90 |

^*^Incudes gender as feature

^**^Combined: acoustic, psycholinguistic, and socio-demographic features

^#^Montreal Cognitive Assessment

HVLT: Hopkins Verbal Learning Test

^$^Animals T-Score (D-KEFS Norms)

^^^FAS Total T-Score (D-KEFS Norms)

**Table S5: Correlation of Geneva minimalistic acoustic parameter set (GeMAPS) acoustic features with age.**

| **Feature name** | **rho** | **p-value** | **significance** |
| --- | --- | --- | --- |
| **F0semitoneFrom27.5Hz sma3nz amean** | 0.079154953 | 0.511718952 |  |
| **F0semitoneFrom27.5Hz sma3nz stddevNorm** | 0.18963246 | 0.113212343 |  |
| **F0semitoneFrom27.5Hz sma3nz percentile20.0** | -0.054841265 | 0.649655716 |  |
| **F0semitoneFrom27.5Hz sma3nz percentile50.0** | 0.057941063 | 0.631257458 |  |
| **F0semitoneFrom27.5Hz sma3nz percentile80.0** | 0.294384825 | 0.012702964 | * |
| **F0semitoneFrom27.5Hz sma3nz pctlrange0-2** | 0.281681066 | 0.017323832 | * |
| **F0semitoneFrom27.5Hz sma3nz meanRisingSlope** | 0.156000128 | 0.193903314 |  |
| **F0semitoneFrom27.5Hz sma3nz stddevRisingSlope** | 0.157688193 | 0.18906378 |  |
| **F0semitoneFrom27.5Hz sma3nz meanFallingSlope** | 0.13540097 | 0.260228293 |  |
| **F0semitoneFrom27.5Hz sma3nz stddevFallingSlope** | 0.104368192 | 0.386390813 |  |
| **loudness sma3 amean** | -0.231012824 | 0.052586212 |  |
| **loudness sma3 stddevNorm** | 0.200854015 | 0.093045157 |  |
| **loudness sma3 percentile20.0** | -0.234195715 | 0.049324916 | * |
| **loudness sma3 percentile50.0** | -0.225805106 | 0.05830084 |  |
| **loudness sma3 percentile80.0** | -0.229305184 | 0.054407311 |  |
| **loudness sma3 pctlrange0-2** | -0.157273082 | 0.190245712 |  |
| **loudness sma3 meanRisingSlope** | -0.146386637 | 0.223164557 |  |
| **loudness sma3 stddevRisingSlope** | -0.086902411 | 0.471143214 |  |
| **loudness sma3 meanFallingSlope** | -0.150187374 | 0.211247146 |  |
| **loudness sma3 stddevFallingSlope** | -0.108916287 | 0.365916552 |  |
| **spectralFlux sma3 amean** | -0.186640066 | 0.119121564 |  |
| **spectralFlux sma3 stddevNorm** | 0.250570106 | 0.035064388 | * |
| **mfcc1 sma3 amean** | -0.145120701 | 0.22723644 |  |
| **mfcc1 sma3 stddevNorm** | 0.121285765 | 0.313666898 |  |
| **mfcc2 sma3 amean** | -0.037481872 | 0.756309266 |  |
| **mfcc2 sma3 stddevNorm** | -0.012765542 | 0.915852612 |  |
| **mfcc3 sma3 amean** | 0.125606669 | 0.296608055 |  |
| **mfcc3 sma3 stddevNorm** | -0.049261186 | 0.683301377 |  |
| **mfcc4 sma3 amean** | 0.189821415 | 0.112846929 |  |
| **mfcc4 sma3 stddevNorm** | 0.21848245 | 0.067179486 |  |
| **jitterLocal sma3nz amean** | 0.042525472 | 0.724744471 |  |
| **jitterLocal sma3nz stddevNorm** | -0.12889889 | 0.284025787 |  |
| **shimmerLocaldB sma3nz amean** | 0.116007001 | 0.335347029 |  |
| **shimmerLocaldB sma3nz stddevNorm** | -0.018775198 | 0.87649952 |  |
| **HNRdBACF sma3nz amean** | 0.030191134 | 0.802636483 |  |
| **HNRdBACF sma3nz stddevNorm** | 0.111406616 | 0.354991892 |  |
| **logRelF0-H1-H2 sma3nz amean** | 0.046994385 | 0.697151731 |  |
| **logRelF0-H1-H2 sma3nz stddevNorm** | -0.114826214 | 0.340322735 |  |
| **logRelF0-H1-A3 sma3nz amean** | 0.045214767 | 0.708095231 |  |
| **logRelF0-H1-A3 sma3nz stddevNorm** | 0.07201314 | 0.550641874 |  |
| **F1frequency sma3nz amean** | 0.15285767 | 0.203148126 |  |
| **F1frequency sma3nz stddevNorm** | 0.142383861 | 0.236215812 |  |
| **F1bandwidth sma3nz amean** | -0.074494567 | 0.536958077 |  |
| **F1bandwidth sma3nz stddevNorm** | 0.143653756 | 0.232019325 |  |
| **F1amplitudeLogRelF0 sma3nz amean** | -0.224760918 | 0.059505283 |  |
| **F1amplitudeLogRelF0 sma3nz stddevNorm** | 0.239367151 | 0.044381198 | * |
| **F2frequency sma3nz amean** | 0.09636851 | 0.424020592 |  |
| **F2frequency sma3nz stddevNorm** | 0.244546669 | 0.039847008 | * |
| **F2bandwidth sma3nz amean** | 0.017090006 | 0.887508772 |  |
| **F2bandwidth sma3nz stddevNorm** | -0.202514521 | 0.090317013 |  |
| **F2amplitudeLogRelF0 sma3nz amean** | -0.221354191 | 0.063575911 |  |
| **F2amplitudeLogRelF0 sma3nz stddevNorm** | 0.233214587 | 0.050312022 |  |
| **F3frequency sma3nz amean** | 0.093757488 | 0.436741641 |  |
| **F3frequency sma3nz stddevNorm** | 0.205624404 | 0.085377293 |  |
| **F3bandwidth sma3nz amean** | -0.15304965 | 0.202574493 |  |
| **F3bandwidth sma3nz stddevNorm** | -0.159186283 | 0.184842367 |  |
| **F3amplitudeLogRelF0 sma3nz amean** | -0.226970735 | 0.056979795 |  |
| **F3amplitudeLogRelF0 sma3nz stddevNorm** | 0.238721035 | 0.044975562 | * |
| **alphaRatioV sma3nz amean** | -0.03225547 | 0.789442497 |  |
| **alphaRatioV sma3nz stddevNorm** | -0.083571117 | 0.488374161 |  |
| **hammarbergIndexV sma3nz amean** | 0.091004811 | 0.450382614 |  |
| **hammarbergIndexV sma3nz stddevNorm** | 0.088784951 | 0.461552933 |  |
| **slopeV0-500 sma3nz amean** | 0.02344384 | 0.846127661 |  |
| **slopeV0-500 sma3nz stddevNorm** | -0.076344413 | 0.526867121 |  |
| **slopeV500-1500 sma3nz amean** | 0.095782127 | 0.426858875 |  |
| **slopeV500-1500 sma3nz stddevNorm** | -0.213319871 | 0.07406991 |  |
| **spectralFluxV sma3nz amean** | -0.149629417 | 0.212967875 |  |
| **spectralFluxV sma3nz stddevNorm** | 0.18482037 | 0.122828896 |  |
| **mfcc1V sma3nz amean** | 0.046103355 | 0.702623453 |  |
| **mfcc1V sma3nz stddevNorm** | 0.113705393 | 0.345088322 |  |
| **mfcc2V sma3nz amean** | -0.071236878 | 0.554956753 |  |
| **mfcc2V sma3nz stddevNorm** | -0.012794583 | 0.915661877 |  |
| **mfcc3V sma3nz amean** | 0.066537286 | 0.581419064 |  |
| **mfcc3V sma3nz stddevNorm** | 0.207942424 | 0.081836271 |  |
| **mfcc4V sma3nz amean** | 0.118319777 | 0.325734788 |  |
| **mfcc4V sma3nz stddevNorm** | -0.144904198 | 0.227937978 |  |
| **alphaRatioUV sma3nz amean** | 0.073062297 | 0.544835877 |  |
| **hammarbergIndexUV sma3nz amean** | -0.048172845 | 0.689938572 |  |
| **slopeUV0-500 sma3nz amean** | 0.030756454 | 0.799017655 |  |
| **slopeUV500-1500 sma3nz amean** | 0.203302178 | 0.089045132 |  |
| **spectralFluxUV sma3nz amean** | -0.181500597 | 0.12981862 |  |
| **loudnessPeaksPerSec** | -0.25544966 | 0.031550395 | * |
| **VoicedSegmentsPerSec** | -0.023202451 | 0.847692941 |  |
| **MeanVoicedSegmentLengthSec** | -0.180506122 | 0.131970165 |  |
| **StddevVoicedSegmentLengthSec** | -0.160913258 | 0.180061125 |  |
| **MeanUnvoicedSegmentLength** | 0.217212961 | 0.068824043 |  |
| **StddevUnvoicedSegmentLength** | 0.223963727 | 0.060438349 |  |
| **equivalentSoundLevel dBp** | -0.162245643 | 0.176434226 |  |

* = <0.05, ** = < 0.01, ***=<0.001

**References**

1. Bruijnen CJ, Dijkstra BA, Walvoort SJ, Budy MJ, Beurmanjer H, De Jong CA, et al. Psychometric properties of the Montreal Cognitive Assessment (MoCA) in healthy participants aged 18–70. International Journal of Psychiatry in Clinical Practice. 2020;24(3):293-300.

2. Jiang L, Xu M, Xia S, Zhu J, Zhou Q, Xu L, et al. Reliability and validity of the electronic version of the Hopkins verbal learning test-revised in middle-aged and elderly Chinese people. Frontiers in Aging Neuroscience. 2023;15:1124731.

3. Delis DC, Kramer JH, Kaplan E, Holdnack J. Reliability and validity of the Delis-Kaplan Executive Function System: an update. Journal of the International Neuropsychological Society. 2004;10(2):301-3.

4. Tyagi N. <https://medium.com/analytics-steps/understanding-the-gini-index-and-information-gain-in-decWoSon-trees-ab4720518ba8>. [cited July 2020]; Available from: <https://medium.com/analytics-steps/understanding-the-gini-index-and-information-gain-in-decision-trees-ab4720518ba8>.

5. S.D. Brown AJM. <https://www.sciencedirect.com/topics/mathematics/gini-index>. Comprehensive Chemometrics , Volume 3. <https://www.sciencedirect.com/topics/mathematics/gini-index2009>. p. GINI Index.

6. Eyben F, Scherer KR, Schuller BW, Sundberg J, André E, Busso C, et al. The Geneva minimalistic acoustic parameter set (GeMAPS) for voice research and affective computing. IEEE transactions on affective computing. 2015;7(2):190-202.

7. Kamiloğlu RG, Boateng G, Balabanova A, Cao C, Sauter DA. Superior communication of positive emotions through nonverbal vocalisations compared to speech prosody. Journal of nonverbal behavior. 2021;45:419-54.

8. Vossebeld F. Towards understanding social interactions through audio signals: University of Twente; 2022.

9. Ishikawa K, Webster J. The formant bandwidth as a measure of vowel intelligibility in dysphonic speech. Journal of Voice. 2020.

10. de Cheveigné A. Formant bandwidth affects the identification of competing vowels. ICPhS, 2093. 1999;2096.

11. Memon SA. Acoustic Correlates of the Voice Qualifiers: A Survey. arXiv preprint arXiv:201015869. 2020.

12. Narasimhan S, Vishal K. Spectral measures of hoarseness in persons with hyperfunctional voice disorder. Journal of Voice. 2017;31(1):57-61.

13. Yamada Y, Shinkawa K, Shimmei K. Atypical Repetition in Daily Conversation on Different Days for Detecting Alzheimer Disease: Evaluation of Phone-Call Data From a Regular Monitoring Service. JMIR mental health. 2020;7(1):e16790.

14. Badal VD, Nebeker C, Shinkawa K, Yamada Y, Rentscher KE, Kim H-C, et al. Do Words Matter? Detecting Social Isolation and Loneliness in Older Adults Using Natural Language Processing. Frontiers in Psychiatry. 2021 2021-November-16;12(728732). doi: 10.3389/fpsyt.2021.728732.

15. Bird S, editor. NLTK: the natural language toolkit. Proceedings of the COLING/ACL 2006 Interactive Presentation Sessions; 2006.

16. Honnibal M, Montani I. spaCy 2: Natural language understanding with Bloom embeddings, convolutional neural networks and incremental parsing. To appear. 2017;7(1):411-20.

17. Miller GA. WordNet: a lexical database for English. Communications of the ACM. 1995;38(11):39-41.

18. Fraser KC, Meltzer JA, Rudzicz F. Linguistic features identify Alzheimer’s disease in narrative speech. Journal of Alzheimer's Disease. 2016;49(2):407-22.

19. Yancheva M, Fraser KC, Rudzicz F, editors. Using linguistic features longitudinally to predict clinical scores for Alzheimer’s disease and related dementias. Proceedings of SLPAT 2015: 6th Workshop on Speech and Language Processing for Assistive Technologies; 2015.

20. Beltrami D, Gagliardi G, Rossini Favretti R, Ghidoni E, Tamburini F, Calzà L. Speech analysis by natural language processing techniques: a possible tool for very early detection of cognitive decline? Frontiers in aging neuroscience. 2018;10:369.

21. Bucks RS, Singh S, Cuerden JM, Wilcock GK. Analysis of spontaneous, conversational speech in dementia of Alzheimer type: Evaluation of an objective technique for analysing lexical performance. Aphasiology. 2000;14(1):71-91.

22. Ahmed S, Haigh AM, de Jager CA, Garrard P. Connected speech as a marker of disease progression in autopsy-proven Alzheimer's disease. Brain. 2013 Dec;136(Pt 12):3727-37. PMID: 24142144. doi: 10.1093/brain/awt269.

23. Jarrold W, Peintner B, Wilkins D, Vergryi D, Richey C, Gorno-Tempini ML, et al., editors. Aided diagnosis of dementia type through computer-based analysis of spontaneous speech. Proceedings of the Workshop on Computational Linguistics and Clinical Psychology: From Linguistic Signal to Clinical Reality; 2014.

24. Boschi V, Catricalà E, Consonni M, Chesi C, Moro A, Cappa SF. Connected Speech in Neurodegenerative Language Disorders: A Review. Front Psychol. 2017;8:269. PMID: 28321196. doi: 10.3389/fpsyg.2017.00269.

25. Khodabakhsh A, Yesil F, Guner E, Demiroglu C. Evaluation of linguistic and prosodic features for detection of Alzheimer’s disease in Turkish conversational speech. EURASIP Journal on Audio, Speech, and Music Processing. 2015;2015(1):1-15.

26. Ash S, Evans E, O'Shea J, Powers J, Boller A, Weinberg D, et al. Differentiating primary progressive aphasias in a brief sample of connected speech. Neurology. 2013;81(4):329-36.

27. Stasak B. An investigation of acoustic, linguistic, and affect based methods for speech depression assessment: UNSW Sydney; 2018.

28. Reilly J, Troche J, Grossman M. Language processing in dementia The Handbook of Alzheimer’s Disease and Other Dementias Budson AE. Kowall NW Wiley-Blackwell. 2011.

29. Orimaye SO, Wong JS-M, Golden KJ, editors. Learning predictive linguistic features for Alzheimer’s disease and related dementias using verbal utterances. Proceedings of the Workshop on Computational Linguistics and Clinical Psychology: From linguistic signal to clinical reality; 2014.

30. Pakhomov S, Chacon D, Wicklund M, Gundel J. Computerized assessment of syntactic complexity in Alzheimer’s disease: A case study of Iris Murdoch’s writing. Behavior research methods. 2011;43:136-44.

31. Yngve VH. A model and an hypothesis for language structure. Proceedings of the American philosophical society. 1960;104(5):444-66.

32. Manning C, Surdeanu, M., Bauer, J., Finkel, J., Bethard, S. and McClosky, D., editor. The Stanford CoreNLP natural language processing toolkit. In  *Proceedings of 52nd annual meeting of the association for computational linguistics: system demonstrations,*ACL; 2014; Baltimore, MD, USA.

33. Nicholas M, Obler LK, Albert ML, Helm-Estabrooks N. Empty speech in Alzheimer's disease and fluent aphasia. Journal of Speech, Language, and Hearing Research. 1985;28(3):405-10.

34. Tomoeda CK, Bayles KA, Trosset MW, Azuma T, McGeagh A. Cross-sectional analysis of Alzheimer disease effects on oral discourse in a picture description task. Alzheimer Disease & Associated Disorders. 1996;10(4):204-15.

35. Masrani V, Murray G, Field T, Carenini G, editors. Detecting dementia through retrospective analysis of routine blog posts by bloggers with dementia. BioNLP 2017; 2017.

36. Scibelli F. Detection of verbal and nonverbal speech features as markers of depression: Results of manual analysis and automatic classification. Napoli: Università degli Studi di Napoli Federico II. 2019.

37. Morales M, Scherer S, Levitan R, editors. A cross-modal review of indicators for depression detection systems. Proceedings of the fourth workshop on computational linguistics and clinical psychology—From linguistic signal to clinical reality; 2017.

38. Hutto C, Gilbert E, editors. Vader: A parsimonious rule-based model for sentiment analysis of social media text. Proceedings of the International AAAI Conference on Web and Social Media; 2014.

39. Ehara Y, editor. Evaluation of unsupervised automatic readability assessors using rank correlations. Proceedings of the 2nd Workshop on Evaluation and Comparison of NLP Systems; 2021.

40. Arnost W, Lull E, Schueder J, Engler J. Reading Level Identification Using Natural Language Processing Techniques. SMU Data Science Review. 2021;5(3):7.

41. Lipton ZC, Elkan C, Naryanaswamy B, editors. Optimal thresholding of classifiers to maximize F1 measure. Machine Learning and Knowledge Discovery in Databases: European Conference, ECML PKDD 2014, Nancy, France, September 15-19, 2014 Proceedings, Part II 14; 2014: Springer.

42. Hicks SA, Strümke I, Thambawita V, Hammou M, Riegler MA, Halvorsen P, et al. On evaluation metrics for medical applications of artificial intelligence. Scientific reports. 2022;12(1):5979.

43. Pandey B, Pandey DK, Mishra BP, Rhmann W. A comprehensive survey of deep learning in the field of medical imaging and medical natural language processing: Challenges and research directions. Journal of King Saud University-Computer and Information Sciences. 2022;34(8):5083-99.

44. Demsar J CT, Erjavec A, Gorup C, Hocevar T, Milutinovic M, Mozina M, Polajnar M, Toplak M, Staric A, Stajdohar M, Umek L, Zagar L, Zbontar J, Zitnik M, Zupan B. Orange: data mining toolbox in Python. *The Journal of Machine Learning Research*  2013;14 (Aug):2349-53.

45. Bengio Y, Grandvalet Y. No unbiased estimator of the variance of k-fold cross-validation. Advances in Neural Information Processing Systems. 2003;16.
